# Does a Starting Positive End-Expiratory Pressure of 8 cmH_2_O Decrease the Probability of a Ventilator-Associated Event?

**DOI:** 10.3389/fmed.2021.744651

**Published:** 2021-11-04

**Authors:** William R. Barnett, Aadil Maqsood, Nithin Kesireddy, Waleed Khokher, Zachary Holtzapple, Fadi A. Safi, Ragheb Assaly

**Affiliations:** ^1^Department of Internal Medicine, University of Toledo, Toledo, OH, United States; ^2^Division of Pulmonary, Critical Care and Sleep Medicine, University of Toledo, Toledo, OH, United States; ^3^College of Medicine and Life Sciences, University of Toledo, Toledo, OH, United States

**Keywords:** ventilator-associated event (VAE), positive expiratory pressure (PEEP), probability model, time between events, quality improvement

## Abstract

**Introduction:** Ventilator-associated events (VAEs) are objective measures as defined by the Centers for Disease Control and Prevention (CDC). To reduce VAEs, some hospitals have started patients on higher baseline positive end-expiratory pressure (PEEP) to avoid triggering VAE criteria due to respiratory fluctuations.

**Methods:** At our institution, VAEs were gathered from January 2014 through December 2019. Using the CDC-defined classifications, VAEs were split into two groups to separate patients with hypoxemia only (VAC) and those with hypoxemia and evidence of inflammation or infection (IVAC-plus). We used the geometric distribution to calculate the daily event probability before and after the protocol implementation. A probability threshold was used to determine if the days between events was exceeded during the post-protocol period.

**Results:** A total of 306 VAEs were collected over the study period. Of those, 155 were VACs and 107 were IVAC-plus events during the pre-protocol period. After implementing the protocol, 24 VACs and 20 IVAC-plus events were reported. There was a non-significant decrease in daily event probabilities in both the VAC and IVAC-plus groups (0.083 vs. 0.068 and 0.057 vs. 0.039, respectively).

**Conclusion:** We concluded a starting PEEP of 8 cmH_2_O is unlikely to be an effective intervention at reducing the probability of a VAE. Until specific guidelines by the CDC are established, hospitals should consider alternative methods to reduce VAEs.

## Introduction

Mechanical ventilation is a life-saving therapy in patients with respiratory failure. Most of these patients carry poor prognosis with a high in-hospital mortality rate (1). Ventilator-associated events (VAEs) are potentially avoidable complications of mechanical ventilation that can lead to longer intensive care unit (ICU) stay, increased risk of morbidity and mortality, and increased healthcare costs. The Centers for Disease Control and Prevention (CDC) developed automatable surveillance algorithms in 2013 to objectively identify nosocomial respiratory conditions including VAEs (2). The CDC defines VAEs by worsening oxygenation status as indicated by increased fraction of inspired oxygen (FiO_2_) or positive end-expiratory pressure (PEEP) after a period of stability. In addition, evidence of inflammation and a positive culture or other laboratory test for respiratory infection are used to determine higher VAE classifications. Using these definitions, VAEs have been classified into three progressive tiers beginning with a ventilator-associated condition (VAC), to an infection-related ventilator-associated complication (IVAC), and then to a possible ventilator-associated pneumonia (PVAP) (2).

In October 2018, our institution implemented a higher baseline PEEP of 8 cmH_2_O (8 PEEP strategy/protocol) from a previous value of 5 cmH_2_O for patients started on mechanical ventilation. The rationale for the change was that an increase in PEEP and not FiO_2_ often preceded a patient meeting the VAC criteria, which started the patient down the VAE spectrum. We exempted neurosurgical patients from the protocol as higher PEEP can increase the intracranial pressure. Likewise, not all patients benefit from a higher PEEP level and thus, our clinicians were not barred from exercising their clinical judgment. The objective of this study was to ascertain whether a starting positive end-expiratory pressure of 8 cmH_2_O is an effective strategy at reducing VAEs in our institution.

## Methods

The study was not deemed as human subjects research by our university's biomedical institutional review board. After being granted exclusion from review, VAEs were gathered from January 2014 to December 2019, which were collected by the Infection Prevention Department as part of daily VAE surveillence. The study population are mechanically ventilated adult patients in the intensive care setting at an academic hospital. For the purpose of this study, we split VAEs into two groups: VAC and IVAC-plus, which is a combination of patients who met either IVAC and/or PVAP criteria.

Rather than relying on traditional measures (events per 1,000 ventilator days) to report changes in the VAE rate, we chose an alternative method to analyze nosocomial infections. More precisely, we used the geometric distribution to calculate a daily probability of a VAE, which is used to determine the likelihood of a rare event occurring over time based on the days between events (3). The hallmark of the geometric distribution is the ability to model a large number of failures (non-VAEs) before a success (VAE) where the probability of an event is the same regardless of how much time has passed. Furthermore, to determine if the probability of an event over time was significantly unexpected (i.e., longer time between events), we used a probability limit of 0.99865. This probability has been used previously as an upper control limit (UCL) as to reduce the likelihood of triggering a false alarm when detecting a change in a nosocomial infection rate (4). The lower control limit (LCL) is typically truncated at zero using this type of methodology.

To graphically depict the VAE cases over time, we used Minitab 17.1 (Minitab LLC, State College, PA) to create charts commonly used in quality improvement to understand variation in healthcare processes.

## Results

**Figure 1:**
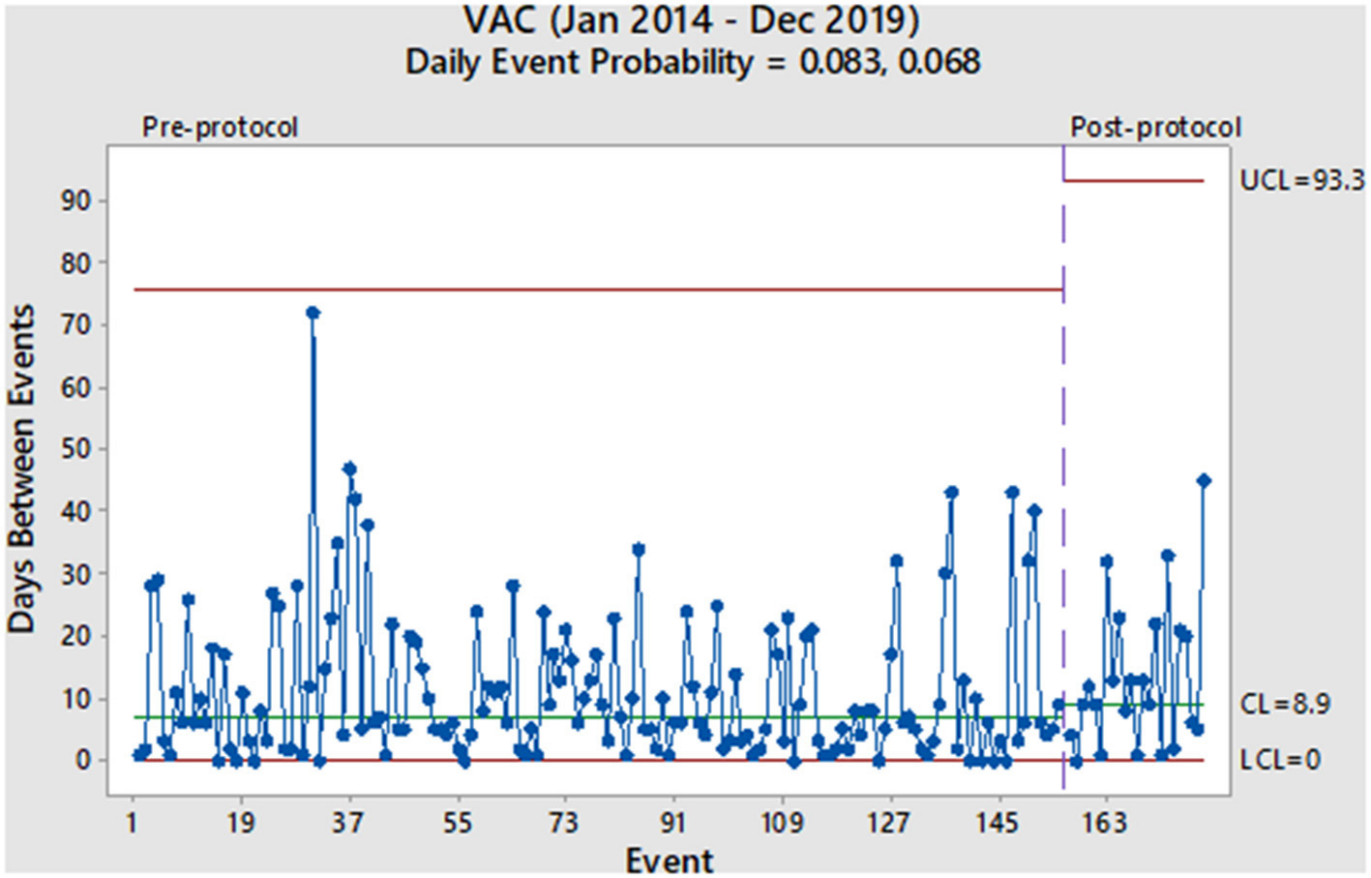
Pre- and post-protocol VAC events (*N* = 179).

**Figure 2:**
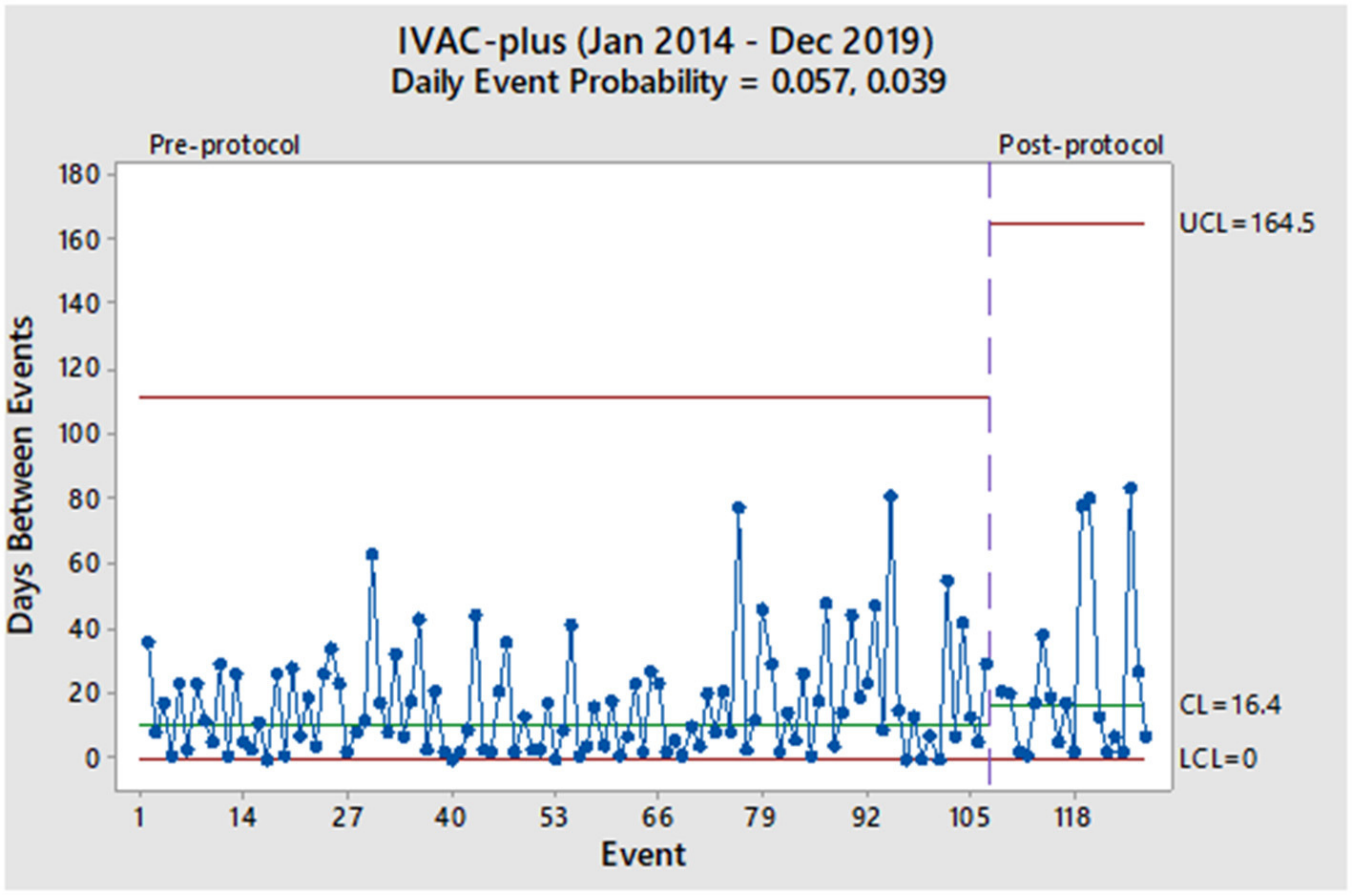
Pre- and post-protocol IVAC-plus events (*N* = 127).

From January 2014 to December 2019, 6,524 mechanically ventilated patients were surveyed (29,568 total ventilator days). During the pre-protocol period, we reported 155 VAC events (7.1/1,000 ventilator days) and 107 IVAC-plus events (4.9/1,000 ventilator days). During the protocol period, we reported 24 VAC events (4.4/1,000 ventilator days) and 20 IVAC-plus events (3.5/1,000 ventilator days). As derived from the geometric distribution, the daily probability of a VAC event decreased from 0.083 to 0.068 without a significant change in the rate (Figure 1). Prior to the protocol, VACs were occurring every 7.0 days based on the centerline (CL) in Figure 1, which denotes the 50th percentile (median) of the geometric distribution corresponding to the daily event probability. After the implementation of the 8 PEEP protocol, the CL showed a modest increase of 8.9 days without a significant event in 15 months. With regards to the IVAC-plus events, the daily probability did decrease from 0.057 to 0.039 without a significant change in the rate (Figure 2). While there was not change in the rate based on the time between events, there was an increase in the CL from 10.8 to 16.4 days.

## Discussion

Given these findings, we would consider the 8 PEEP strategy to not have a significant impact in decreasing the probability of a VAE. More specifically, both charts show random variation over time and without a significant event (i.e., more than 93 days between VACs and more than 164 days between IVAC-plus). Also, it has been more than a year after protocol implementation and would be unlikely that any subsequent change in the VAE rate at this point would be due to the 8 PEEP protocol. However, the increase in the median time between events among IVAC-plus patients could represent a clinically important outcome where cases were slightly reduced. We theorize a higher PEEP strategy could potentially be beneficial to patients with non-infectious etiologies of VAEs, such as those due to atelectasis and pulmonary edema. This finding was based on evidence that some cases of VAC had improved and were likely not moving onto the IVAC-plus category.

Aside from our evaluation of the 8 PEEP strategy, our overall impression of the two charts is that VAEs occur in our institution with a high rate of variability throughout the pre- and post-intervention periods. Furthermore, this phenomenon continues to occur in spite of other interventions used in our ICU, such as a ventilator-associated pneumonia (VAP) prevention bundle (oral care, head of bed elevation, spontaneous breathing trials, etc.) and any additional concerted quality improvement efforts to reduce VAEs. While we observe some longer stretches of time between events, such as 1–2 months, these are non-significant random changes and do not represent true changes in the VAE rate.

While higher PEEP is theorized to reduce VAE trigger caused by non-infectious etiology like heart failure and atelectasis, it may have counterproductive effects on vascular physiology. Increasing PEEP can cause proportional decrease in cardiac output *via* several mechanisms. The most important being decreased venous return to the right heart, due to elevated intrathoracic pressure. This in turn leads to reduced cardiac output and may lead to worsened hypotension especially in patients with volume depletion and right sided heart failure (5, 6). Secondly, increasing PEEP can potentially increase pulmonary vascular resistance due to distension of alveoli and collapse of alveolar vessels (6). Positive intrathoracic pressure is transmitted to pulmonary arteries leading to increase in right ventricular afterload as well. The increasing right ventricular pressure can cause leftward displacement of the interventricular septum resulting in decreased left ventricular filling and adversely affect cardiac output (7). Higher PEEP has been shown to decrease cerebral blood flow (CBF), but this generally occurs in patients with impaired cerebrovascular autoregulation (8). Patients with intracranial hemorrhages or traumatic brain injuries can suffer from impaired cerebrovascular autoregulation, leading to mean arterial pressure dependent cerebral perfusion. In addition, intracranial hemorrhages can cause increased intracranial pressures (ICP) (8).

The limitations to these findings are that the study was conducted retrospectively and at a single center. In addition, there are other factors that can potentially influence changes in the ventilator settings, which were not explored. Also, we did not study other risk factors that have been implicated in VAE development, such as deep sedation, fluid overload, and high tidal volume. Moreover, certain treatments, such as fluid resuscitation protocols and lung protective strategy, were assumed to be present before and after implementation. Despite these shortcomings, the pre-protocol probabilities were calculated on 4-1/2 years of data and the post-protocol period was ample time to await any change in the VAE rate. Future studies are required to sufficiently assess the effect of higher baseline PEEP on reducing VAE development.

Since the implementation of VAE surveillance programs by the CDC, some hospitals have adopted policies to raise baseline PEEP to avoid triggering VAC criteria with respiratory fluctuation as a potential method to reduce VAEs (9, 10). Our experience with using an 8 PEEP protocol was it does not appear to be a long-term solution to reduce VAEs. Rather than relying on these types of strategies, we suggest rigorous measurement of a hospital's VAE rate and adopting specific interventions to effect change. Without clear definitions and guidelines from the CDC on preventing VAEs, the system remains prone to manipulation (11–13).

## Data Availability Statement

The raw data supporting the conclusions of this article will be made available by the authors, without undue reservation.

## Author Contributions

RA conceived and designed the study. WB was guarantor of this work, had full access to all the data in the study, takes responsibility for its integrity, the accuracy of the data analysis participated in the acquisition of data, and analyzed the data. AM, NK, WK, and ZH drafted the manuscript. WB, FS, and RA revised the manuscript. All authors have read and approved the final manuscript.

## Conflict of Interest

The authors declare that the research was conducted in the absence of any commercial or financial relationships that could be construed as a potential conflict of interest.

## Publisher's Note

All claims expressed in this article are solely those of the authors and do not necessarily represent those of their affiliated organizations, or those of the publisher, the editors and the reviewers. Any product that may be evaluated in this article, or claim that may be made by its manufacturer, is not guaranteed or endorsed by the publisher.
